# Estimation of Strawberry Canopy Volume in Unmanned Aerial Vehicle RGB Imagery Using an Object Detection-Based Convolutional Neural Network

**DOI:** 10.3390/s24216920

**Published:** 2024-10-28

**Authors:** Min-Seok Gang, Thanyachanok Sutthanonkul, Won Suk Lee, Shiyu Liu, Hak-Jin Kim

**Affiliations:** 1 Department of Biosystems Engineering, College of Agriculture and Life Sciences, Seoul National University, Seoul 08826, Republic of Korea; msg1907@snu.ac.kr; 2 Integrated Major in Global Smart Farm, College of Agriculture and Life Sciences, Seoul National University, Seoul 08826, Republic of Korea; 3 Department of Agricultural & Biological Engineering, University of Florida, Gainesville, FL 32611, USAshiyu.liu@ufl.edu (S.L.); 4 Research Institute of Agriculture and Life Sciences, Seoul National University, Seoul 08826, Republic of Korea

**Keywords:** canopy volume, growth estimation, UAV, YOLO, RGB images

## Abstract

Estimating canopy volumes of strawberry plants can be useful for predicting yields and establishing advanced management plans. Therefore, this study evaluated the spatial variability of strawberry canopy volumes using a ResNet50V2-based convolutional neural network (CNN) model trained with RGB images acquired through manual unmanned aerial vehicle (UAV) flights equipped with a digital color camera. A preprocessing method based on the You Only Look Once v8 Nano (YOLOv8n) object detection model was applied to correct image distortions influenced by fluctuating flight altitude under a manual maneuver. The CNN model was trained using actual canopy volumes measured using a cylindrical case and small expanded polystyrene (EPS) balls to account for internal plant spaces. Estimated canopy volumes using the CNN with flight altitude compensation closely matched the canopy volumes measured with EPS balls (nearly 1:1 relationship). The model achieved a slope, coefficient of determination (R^2^), and root mean squared error (RMSE) of 0.98, 0.98, and 74.3 cm^3^, respectively, corresponding to an 84% improvement over the conventional paraboloid shape approximation. In the application tests, the canopy volume map of the entire strawberry field was generated, highlighting the spatial variability of the plant’s canopy volumes, which is crucial for implementing site-specific management of strawberry crops.

## 1. Introduction

Strawberries are among the most valuable crops worldwide. However, early scheduling of labor is challenging as a result of weekly harvest variations influenced by various factors, including photoperiod and temperature fluctuations [1]. Furthermore, failure to harvest ripe strawberry fruits, on time due to labor shortages can lead to severe economic losses. In order to avoid such wastage, reliable yield prediction and planning of labor needs are required in advance for harvesting [2,3].

Traditional yield estimation methods, which involve manual crop sampling, are time-consuming and labor-intensive. Phenotypic characteristics, such as canopy volume, crown area, and canopy surface area, can facilitate yield prediction and field management. Accurate measurement of canopy volume is particularly crucial for monitoring crop growth and estimating the potential yield in horticulture [4,5,6,7,8]. Since canopy volume serves as an early indicator of drought and fertilization stress in plants, yield fluctuations caused by these factors can be identified in advance [9].

Zhu et al. [10] assessed various techniques for measuring the crown volume, including simple geometric shape approximations, computational geometry approaches, and voxelization methods. The differences between crown and canopy volumes may be less significant in widely spaced plants [11,12], and the term ‘crown volume’ has been often replaced by ‘canopy volume’ [10]. Conventionally, canopy volume is determined by measuring the canopy diameter and canopy height manually and approximating the shape with solid geometric objects, such as elliptical cylinders, cuboids, cones, and ellipsoids [13,14,15,16]. To reduce acquisition time, some studies have introduced ultrasonic, laser, and light detection and ranging (LIDAR) sensors for automatic canopy diameter and height measurement [17,18,19,20]. Computational geometric approaches utilize digital data, such as three-dimensional (3D) point clouds, RGB-D imagery, and data fusion [10], to estimate crop growth. These data can be acquired using ground or airborne LIDAR- and camera-based techniques. Canopy volume can be represented as a 3D convex hull [21] enclosing the entire canopy as a convex polygon [22,23,24]. Several studies [23,25] have also estimated canopy volumes using alpha shapes [26] based on Delaunay triangulation or using canopy height models from raster data, where the volume is obtained by multiplying the area and height of each grid cell and summing them [7]. Meanwhile, the voxelization technique divides the 3D space occupied by the canopy into small volumetric pixels [27] and estimates the canopy volume by summing these voxels [5,24,28,29].

Despite such advancements, canopy volume estimation based on geometric object approximation, computational geometry, and voxelization remains time-consuming and involves complex calculations, costly equipment, or powerful computers to process and analyze canopy structure data, especially 3D information. Moreover, intense research is still needed to estimate canopy volumes accurately while excluding internal voids. The complex structures and irregular canopy shapes make precise canopy volume measurements using simple geometric methods challenging. Furthermore, 3D convex hulls are limited in reconstructing irregular cross sections, while alpha shapes are known to represent internal empty spaces poorly. Apart from that, grid cell-based volume calculations can be inaccurate when processing complex 3D structures or non-convex shapes. Voxel techniques may also struggle to accurately estimate internal volumes without measuring points inside densely packed leaves [30,31]. Additionally, the use of ranging sensors is more difficult for low-height crops, such as strawberries, due to constraints on measurement angles, interference from environmental obstacles, and difficulties with sensor placement.

Recently, several advanced studies have revealed the effective use of deep learning regression models for estimating crop growth indicators. Deep neural networks offer an edge in solving nonlinear problems and provide relatively faster inference speeds over traditional computer-vision methods. Specifically, convolutional neural networks (CNNs) are effective in learning complex and abstract image features through a two-dimensional (2D) array of kernels. These kernels identify various patterns and textures, extracting valuable information from the images [32]. For example, Ma et al. employed a CNN to predict the above-ground biomass of winter wheat during early growth stages, with a strong coefficient of determination (R^2^) of 0.8 [33]. In another study, Gang et al. developed a two-stage CNN model for RGB-D images of greenhouse lettuce with concurrent estimation of fresh weight, dry weight, height, diameter, and leaf area as the growth indices. The findings recorded high R^2^ values of 0.95, 0.95, 0.95, 0.88, and 0.96, respectively [34].

The advantages of deep learning regression models can be further boosted by integration with unmanned aerial vehicle (UAV) imagery. UAV images have been extensively employed in the agricultural sector for monitoring and estimating crop growth metrics, including crop volume [3,13,22,35,36,37,38]. UAVs can also be used to survey large agricultural areas effectively and provide growers with essential data in a timely manner. In addition, UAVs can be equipped with high-resolution cameras and fly at low altitudes to capture detailed characteristics of plants, enabling precise individualized monitoring and crop management [39]. Previously, Castro et al. utilized a CNN model with UAV RGB images for forage biomass estimation and achieved an R^2^ of 0.88 [40]. Liu et al. combined UAV RGB images with thermal-infrared (TIR) images using a deep neural network (DNN) to estimate the leaf area index (LAI) of maize, which also recorded a high R^2^ value of 0.89 [41]. Likewise, Zheng et al. estimated the canopy leaf area and dry biomass of strawberry plants by measuring the plant’s canopy area, volume, and height using a CNN with RGB, near-infrared (NIR), a digital surface model (DSM), and mask images extracted from UAV images, yielding robust R^2^ values of 0.986, 0.927, and 0.847, respectively [7,42]. Nevertheless, the target volume used for training was obtained from a grid cell-based calculation, which may vary from the actual canopy volume. In addition, canopy volume was estimated only for specific segments of the field. Therefore, canopy volume estimation using deep learning models, such as CNNs, still remains a challenge. Reducing the number of sensors is also vital to ensure cost-effectiveness and increase their applicability to agricultural tasks.

Furthermore, the use of autonomous flight systems based on Global Navigation Satellite System (GNSS) information requires strict regulations and certification procedures, which pose a significant hurdle in UAV research. The successful completion of UAV missions can be hampered by GNSS signal interference and obstacles. Moreover, the sensors and software related to autonomous flight systems are costly [43].

While UAV image registration methods based on feature points and cross-correlation of images are applicable to adjusting altitude and position in manual flights [44,45,46], such approaches are computationally intensive. In addition, feature-based registration may struggle to extract valid points in complex vegetation scenes, and area-based registration may be sensitive to geometric distortions and noise, leading to incorrect alignment. In view of this, a faster and more fail-safe method for image correction and field image synthesis method is needed for agricultural applications using manually flown UAVs.

Therefore, this study developed a CNN model based on only RGB images obtained with a UAV operated in manual mode for rapid and accurate prediction of strawberry canopy volumes. The accuracy of the CNN model was compared to a linear regression model based on conventional simple geometry using a paraboloid. Image preprocessing and image synthesis methods based on object detection were designed using the You Only Look Once (YOLO) algorithm to compensate for the changes in altitude during manual flight and to match RGB images without the GNSS information to generate entire row images. Finally, the developed CNN model was applied to the row images to construct a map displaying spatial variability in canopy volumes at varying locations in a field.

## 2. Materials and Methods

### 2.1. Setup of Strawberry Field Experiment

A strawberry experimental field was prepared in the Plant Science Research and Education Unit (PSREU) at the University of Florida in Citra, Florida, USA, during the 2023–2024 growing season. The field comprised eight rows of strawberry plants and two rows of sprinklers, with an approximate total size of 61 m long and 20.7 m wide. Four rows of the ‘Brilliance’ cultivar were planted in the northern half of the field, while the other four rows in the southern half consisted of the ‘Medallion’ cultivar. The ‘Brilliance’ cultivar is characterized by its broad-spread leaves [47], in contrast to the more compact and upright structure of the ‘Medallion’ cultivar [48]. Each row contained two alternating lines of strawberry plants. The overripe fruits, runners, dead leaves, and weeds were removed regularly.

### 2.2. Acquisition of UAV-Based RGB Images

**Figure 1:**
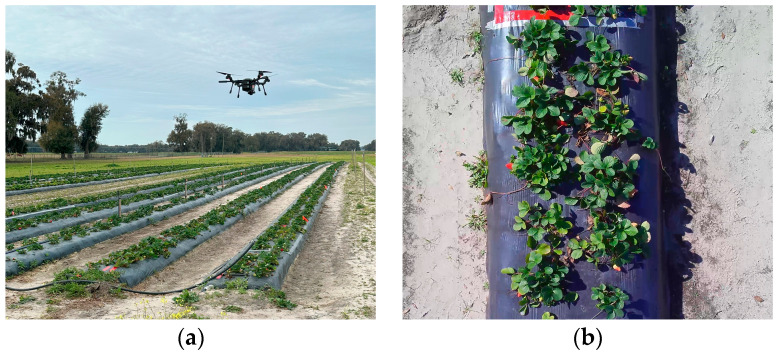
(**a**) Manual UAV flight over the strawberry experimental field. (**b**) RGB images at a resolution of 1920 × 1080 pixels were extracted and resized from the UAV video frames captured during manual UAV flight.

This study employed a SIRAS drone (Teledyne FLIR LLC., Wilsonville, OR, USA) to capture RGB MOV videos of each strawberry row (Figure 1a). The SIRAS weighs 3.1 kg and exhibits a flight time of 31 min. It was equipped with a 16-megapixel RGB camera featuring an aperture with a F-number of 2.3, a 4.8 mm focal length lens providing a 67° Field of View (FOV), and a maximum video resolution of 4K at 30 frames per second. The video in this study was recorded in 3840 × 2160 pixels. Video snapshots were extracted every 10 frames to construct a dataset for the CNN and resized to 1920 × 1080 pixels, as shown in Figure 1b for use in the object detection model. The images were captured once per week over four weeks from 2 to 23 February 2024, during the harvest season, which runs from December through March in Florida [3]. The drone was flown manually at an approximate speed of 1 m/s within a flight altitude range of 2.5–4 m without GNSS assistance.

Five strawberry plants per row were selected from a total of eight rows for manual investigation of the canopy volumes, plant widths, lengths, and heights, which totaled 40 plants. The sample plants were marked with red and yellow tapes to distinguish between the Brilliance and Medallion varieties, respectively, for easy identification for weekly measurement. Afterward, approximately 984 images containing the sampled plants were selected for the CNN model training, where six to seven images were taken for each sample plant at different angles by adjusting the video frames. The diverse images were expected to expand the dataset size and enhance the estimation accuracy of the CNN.

### 2.3. Image Preprocessing for the Development of CNN Model

#### 2.3.1. Resizing UAV Images Using YOLOv8n to Correct Flight Altitude Deviations from Manual Flight

Image preprocessing was performed using OpenCV and Python to build a dataset for training the model in estimating the canopy volumes of individual strawberry plants. To compensate for the changes in flight height due to manual flight control, the images were resized according to the number of visible plants and missing plants in each UAV image.

First, the number of plants and missing plants in each image were detected and counted using the YOLO v8 Nano (YOLOv8n) object detection deep learning model. YOLO has been extensively applied for effective and efficient object detection in various applications, including agriculture, due to its real-time processing, exceptional accuracy, and ease of implementation [49]. YOLOv8 effectively balances detection speed and performance, and YOLOv8n is the most compact model in the YOLOv8 series. Furthermore, YOLOv8n reduces the level of computation compared to other models, rendering it suitable for detection tasks involving fewer categories and simpler functions [50].

**Figure 2:**
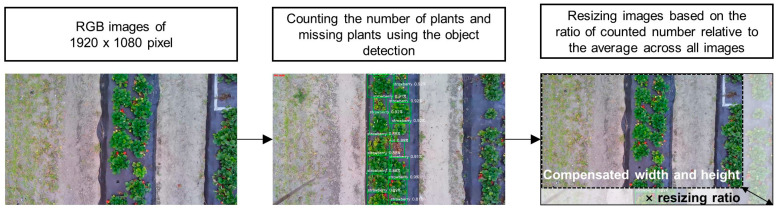
A flowchart of the object detection-based image preprocessing to resize the images using the ratio of the number of counted plants and missing plants to the average number of plants and missing plants across all images (Equations (1)–(3)).

Prior to the YOLOv8n model training, the LabelImg Python open-source tool was utilized to manually label strawberry plants, missing plants, missing plants with weeds, and dead plants in each 1920 × 1080-pixel image (approximately 2549.76 megabytes). Then, the model was pre-trained using the COCO128 dataset [51,52]. Hyperparameters were set to the same preset values as those used for the pre-trained model, including an AdamW optimizer [53] with a learning rate of 0.001111, momentum of 0.9, and decay of 0.0005 for 64 weights. Training was conducted for 50 epochs on a desktop computer running Windows 10 (Microsoft, Redmond, WA, USA) installed with an Intel i7-11700 Central Processing Unit (CPU) (Intel, Santa Clara, CA, USA), a GeForce RTX 3090 Graphics Processing Unit (GPU) (NVIDIA, Santa Clara, CA, USA), and 128 GB of RAM. The model was implemented using the PyTorch 2.0.1 framework and Python 3.9. The YOLOv8n dataset consisted of snapshots including the sampled plants and the dataset contained 984 images comprising 689 images (70%) for training and 295 images for testing. The total number of parameters in the object detection model was 3,157,200, and the estimated size of the model based on the number of parameters was 12.63 megabytes. Once YOLOv8n was applied to the images, the ratio of the number of counted plants and missing plants to the average number of plants and missing plants across all images was calculated (Equation (1)) to determine the relative difference in shooting height. The image size was then multiplied by the resizing ratio (Equations (2) and (3)) to ensure consistent plant size, regardless of the flight altitude. Figure 2 describes the process of the presented image preprocessing.

(1) $$Resizing\;ratio=\frac{Number\;of\;plants\;and\;missing\;plants\;in\;each\;image}{Average\;number\;of\;plants\;and\;missing\;plants}$$

(2) Compensated image width=Resizing ratio×Image width

(3) Compensated image height=Resizing ratio×Image height

**Figure 3:**
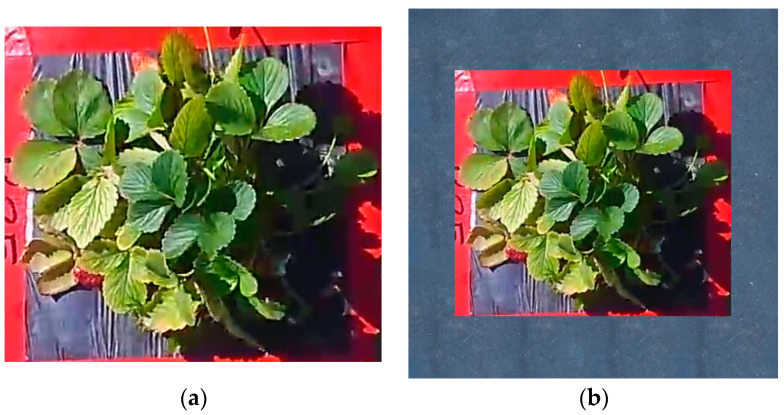
(**a**) Cropped and resized ROI image of individual sample plants. (**b**) Sample plant images centered on a 512 × 512-pixel mulch background.

Subsequently, regions of interest (ROIs) were cropped manually from the images to focus on the sampled individual plants. The final dataset comprised resized sample plant images, each centered on a 512 × 512-pixel background for CNN processing (Figure 3). The background was generated using a randomly selected mulch texture.

#### 2.3.2. Evaluation of the YOLOv8n Object Detection Model

The performance of the object detection model was evaluated by examining key metrics, including precision, recall, F1-score, mean average precision at the intersection over union (IoU) 50% (mAP50), and average frames per second (AVGFPS), similar to the methods employed in previous YOLO studies [54]. The target confidence threshold was set at 0.65, and the intersection over union (IoU) value during the test was fixed at 0.7. The precision, recall, and F1-scores were computed using Equations (4)–(6), respectively. The frames per second and mAP50 values were measured using the validation functions integrated into YOLOv8n.

(4) $$Precison\;(\%)=\frac{TP}{TP+FP}\times 100$$

(5) $$Recall\;(\%)=\frac{TP}{TP+FN}\times 100$$

(6) $$F1\;score=\frac{2\times Precison\times Recall}{Precison+Recall}$$

where *TP*, *FP*, and *FN* refer to the number of true positive, false positive, and false negative cases, respectively.

### 2.4. Development of CNN Model for Canopy Volume Estimation

#### 2.4.1. Construction and Training of CNN Model

**Figure 4:**
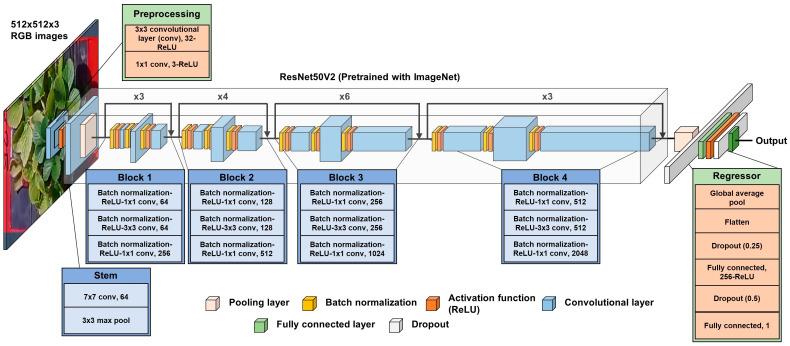
Description of the CNN model for estimating canopy volume, adopted from Gang et al. [34] with modifications. The model utilizes a pre-trained ResNet50V2 [55] with ImageNet [56] as the backbone, with two convolutional layers used for preprocessing, followed by a fully connected layer for regression.

The CNN model was developed to estimate canopy volumes from input RGB images with a resolution of 512 × 512 pixels. As shown in Figure 4, the CNN structure was designed to accept only RGB images as inputs and generate single volume outputs, differing from the two-stage CNN model used for growth index estimation with RGB-D images, as presented by Gang et al. [34]. The model architecture was developed using the TensorFlow 2.5.1 framework and Python 3.8.

ResNet50V2 [55], an improved version of ResNet50 [57] and pre-trained using the ImageNet dataset [56], was utilized as the backbone. Basically, ResNet is able to solve the gradient vanishing problem by adding residual blocks and a skip connection. It is also equipped with the activation function before the convolutional layer in the residual block, which further improves its performance [55,57].

As in the training of the object detection model, the dataset consisted of 984 images, with 689 images (approximately 70%) for training and 295 images for testing. The model was trained on a desktop computer, and the same one was used to train the YOLOv8n object detection model. The hyperparameter settings were set similar to those applied in a previous study [34]. The initial learning rate was fixed at 0.001, and training was conducted over 200 epochs. A learning rate scheduler was used to halve the learning rate if validation loss did not decrease after eight iterations. Early stopping was applied to minimize unnecessary training time if validation loss remained unchanged after 30 iterations. The input batch size was set at 32, and the model weights were retained when validation loss reached its lowest value. Furthermore, five-fold cross-validation was applied to prevent overfitting due to fixation to the validation dataset [58]. The total number of parameters in the developed model was 24,099,844, and the estimated size of the model based on the number of parameters was 91.93 megabytes.

#### 2.4.2. Manual Measurement of Canopy Volume Used as Target Values for CNN

**Figure 5:**
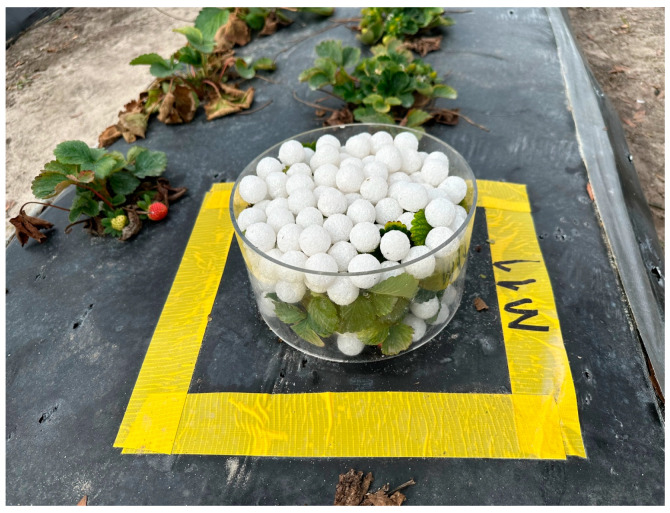
An acrylic cylindrical case with a strawberry plant filled with EPS balls to calculate the volume difference between the number of balls with and without plants.

The actual canopy volumes for each strawberry plant grown in a field were measured through several steps. First, an acrylic cylindrical case (internal diameter of 19.4 cm and a height of 10.1 cm) was placed on a flat surface over the plastic mulch without the plant. The case was filled with small expanded polystyrene (EPS) balls (diameter of 2 cm), and the number of balls was manually counted (Table 1). Next, the case was placed over a strawberry plant, excluding the fruit and dry leaves, before being filled with EPS balls up to the top of the cylinder. The number of balls was again counted (Figure 5). The difference in the number of balls between the empty case and the case filled with the plant was used to measure the canopy volume (Equations (7) and (8)). Extra care was given when placing the balls into the case to avoid damaging the plant leaves and to ensure that the maximum height of the EPS balls did not exceed the height of the case. The offset per ball was calculated (Equation (8)) by dividing the gap between the case and the balls by the total number of EPS balls (240 pieces), as shown in Table 1.

(7) $$Canopy\;volume=\left(Volume\;of\;a\;ball+Offset\;per\;ball\right)\times Difference\;in\;the\;number\;of\;balls$$

(8) $$Offset\;per\;ball=\frac{Case\;volume-(Volume\;of\;a\;ball\times Total\;number\;of\;balls)}{Total\;number\;of\;balls}$$

#### 2.4.3. Manual Measurement of Canopy Fullness Level Used as Target Values for CNN

Typically, canopy fullness refers to the percentage of canopy area occupied by leaves [59,60] and is closely associated with canopy volume. In this study, canopy fullness was assessed as an alternative variable to the target values for training the canopy volume estimation model. Canopy fullness of the sample plants was measured on a scale from zero to ten based on manual observation, with an average observation time of roughly 15 min for 40 samples.

A maximum canopy fullness level denotes the condition when the acrylic case used for the EPS ball-based canopy volume measurement is 100% filled with canopy, matching the volume of the acrylic case. Hence, this fullness level can be converted to an approximate canopy volume (Equation (9)) and was used as the target value for training the CNN model.

(9) $$Canopy\;volume\;converted\;from\;canopy\;fullness\;level\;=Volume\;of\;the\;acrylic\;case\;\;\;\;\;\;\;\times \left(\frac{Canopy\;fullness\;level\times Maximum\;level}{100}\right)$$

#### 2.4.4. Mixture of Manually Measured Canopy Fullness and Canopy Volume Used as Target Values for CNN

Three models were trained to assess the effectiveness of fine-tuning the models by canopy fullness level with canopy volumes converted from the canopy fullness level. The first model was trained with 100% of the canopy volume measured using the EPS balls, while the second model was trained with 100% of the canopy volume converted from the canopy fullness level. The third model was trained by mixing 50% of the canopy volume converted from canopy fullness level with 50% of the canopy volume based on EPS balls.

#### 2.4.5. Evaluation of the Developed Estimation Model

The canopy volumes were estimated using a test dataset that was not used for training. The following procedures describe the estimation accuracy evaluation of the developed model over four weeks through the R^2^ and root mean squared error (RMSE) analysis.

(1) The performance of the developed CNN model was evaluated by comparing the R^2^ and RMSE values from a linear regression model based on a geometric shape approximation. The paraboloid shape, which is the conventional geometric shape used in canopy volume estimations, was used in this study for canopy volume estimation [10]. The linear regression model was established between the measured canopy volumes using EPS balls and the volumes of paraboloids, calculated based on the widths, lengths, and heights of the strawberry plants, using the same dataset as the CNN model. The formula for calculating the volume of a paraboloid is expressed in Equation (10). Subsequently, the regression model was applied to the test set.

(10) $$Paraboloid:V_{p}=\frac{\pi \cdot d^{2}\cdot h}{8}$$

where $V_{p}$ refers to the canopy volume, d represents the plant diameter, and h denotes the plant height.

(2) The impact of the proposed image preprocessing method for correcting flight altitude deviations in the manual flight was examined by comparing the R^2^ and RMSE values of the canopy volumes estimated from the developed CNN model with those measured using EPS balls, both before and after image preprocessing.

(3) The performance of the CNN model for canopy volume estimation based on varying target values was assessed by comparing the following: (i) canopy volumes measured using EPS balls, (ii) canopy volumes converted from canopy fullness level, and (iii) canopy volumes by mixing 50% of converted canopy volume with 50% of canopy volume based on EPS balls as target values.

### 2.5. Generation of Canopy Volume Distribution Maps

The practical application of the developed CNN model was assessed by constructing maps to obtain an approximate spatial distribution of canopy volume over the entire field without GNSS information. Images of each crop row were first synthesized, followed by extraction of individual plant images using YOLOv8n, processing these images with the CNN to estimate canopy volumes, and finally interpolating the results.

Video snapshots of 1920 × 1080 pixels for each frame from the weekly RGB video recorded by the UAV without GNSS information were extracted. Then, images of an entire row were automatically assembled by cutting overlapped areas between the snapshots and pasting the cropped snapshots in frame sequences. The size of each cropped snapshot was determined using values derived from the average optical flow magnitudes, which were calculated using OpenCV between binary images marking the center of the bounding boxes detected using the YOLOv8n object detection model, as described in Section 2.3.

Afterward, the synthesized row images were once again divided into 1920 × 1080-pixel sections, and fed into the object detection model for individual plant extraction. Next, the extracted plant images were preprocessed and input into the developed CNN model for further analysis.

**Figure 6:**
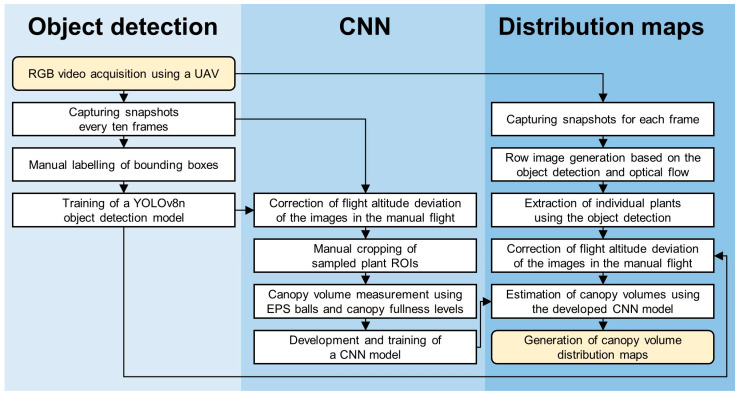
An overview of the development and testing process for estimating strawberry canopy volume in UAV RGB imagery using an object detection-based CNN.

The Cartesian coordinates of each detected strawberry image’s volume data were automatically aligned using the center coordinates of the bounding box. These coordinates were normalized based on the actual row spacing and inter-crop spacing. Finally, a virtual growth map was constructed using the QGIS open-source tool based on the Universal Transverse Mercator. Ordinary kriging built into QGIS was applied to estimate the overall growth distribution and interpolate canopy volumes in areas where object detection failed. The cell size was set to half the average distance between data points, with the number of lag distance classes set to 15% of the maximum field experiment distance. The block size was set to approximately 1.5 times the cell size. Figure 6 briefly outlines the overall process of this study.

## 3. Results

### 3.1. Performance of Object Detection Model

Table 2 summarizes the model performance of the object detection model. Accordingly, the detection accuracy for missing plants with dead plants and weeds was slightly lower, possibly due to the unbalanced number of ROI images for strawberries and missing plants. Given the 94.3% detection rate of labeled bounding boxes, regardless of true or false, the object detection model indicated satisfactory performance for applying flight height correction to the images.

### 3.2. Canopy Volume Estimation Using CNN

#### 3.2.1. Comparison of R^2^ and RMSE Values Between Linear Regression Model Using Paraboloid Shape and CNN Model

**Figure 7:**
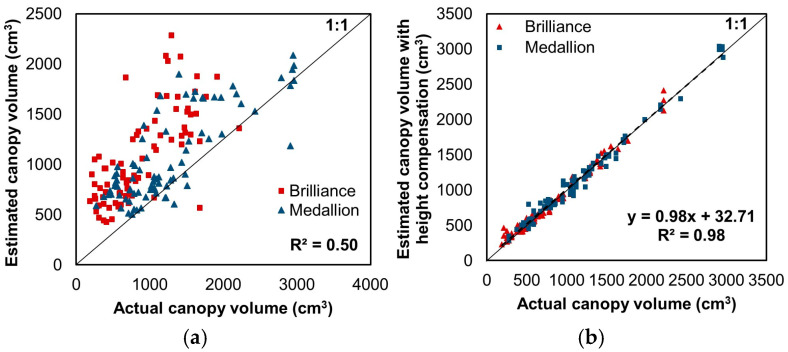
(**a**) Comparison of canopy volumes estimated from the linear regression model using a paraboloid shape and the actual canopy volumes measured using EPS balls. (**b**) Comparison of canopy volumes estimated from the developed model using RGB test dataset images and measured canopy volumes with height compensation using the object detection model. The color symbols represent the values for each strawberry variety. The dashed line shows the regression line.

Figure 7a presents the comparison of the canopy volumes estimated from the paraboloid shape using the linear regression model and the actual volume measured using EPS balls. The linear regression model revealed a coefficient of the input variable and the y-intercept of the paraboloid volume of approximately 0.2 and 321.1, respectively. The R^2^ and RMSE between the estimated canopy volume and the actual measured volume were 0.5 and 462.2 cm^3^, respectively. Meanwhile, Figure 7b shows the canopy volume estimation conducted using the CNN model with image preprocessing based on the object detection model. The R^2^ was 0.98, with an RMSE of 74.4 cm^3^, which was 4.2 cm when converted to cubic root dimensions. This corresponds to the volume of a small golf ball-sized cube.

The R^2^ and RMSE of the Brilliance variety were 0.98 and 68.5 cm^3^, and those of the Medallion variety were 0.98 and 73.7 cm^3^, respectively. Because the maximum Brilliance canopy volume for the test dataset was smaller than that of Medallion, the RMSE of Brilliance was slightly lower. Nevertheless, the overall volume prediction performance of the developed model was similar for both varieties and deemed acceptable considering the underlying factors, such as variations in altitude and shooting angles resulting from the manual UAV flight, the presence of weeds in the field, occlusion between plants, and potential errors in counting the EPS balls. In short, the developed model displayed significantly improved performance compared to the volume prediction using the paraboloid. In addition, the prediction speed of the developed CNN model was 0.05 s per image in the environment described in Section 2.3.

#### 3.2.2. Canopy Volume Estimation Using EPS Ball-Based Target Values Before and After Correction of Flight Altitude Deviations

**Figure 8:**
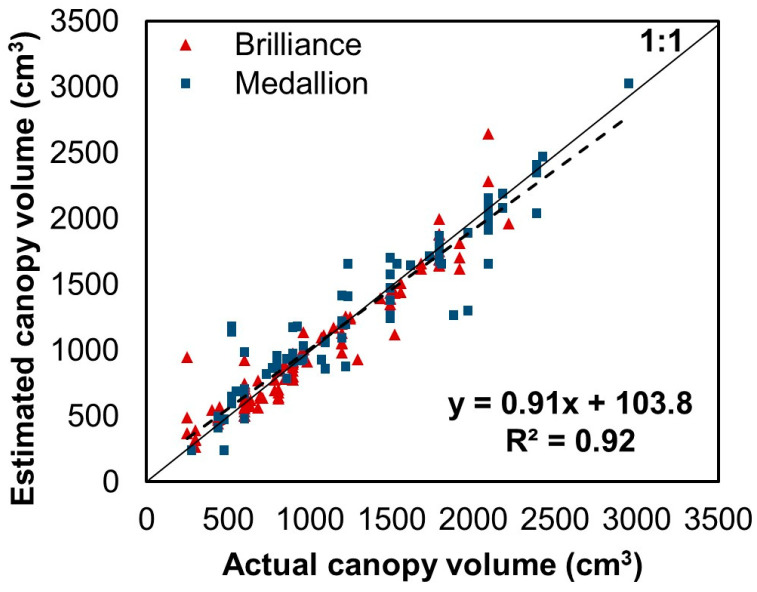
Canopy volumes estimated from the developed model using RGB test dataset images and measured canopy volumes without height compensation using the object detection model. The color symbols indicate values for each strawberry variety. The dashed line shows the regression line.

Figure 8 shows the canopy volume estimation conducted using the CNN model without image preprocessing based on the object detection model. Upon application of the CNN model to the test image set, the R^2^ and RMSE were 0.92 and 167.6 cm^3^, respectively. In contrast, when the proposed image preprocessing was applied to the CNN model, the R^2^ showed a higher linearity, as shown in Figure 7b, and RMSE decreased by 55.6%. This result demonstrates that the proposed image preprocessing method effectively compensated for the difference in image capture height due to altitude deviations in the manual flight.

#### 3.2.3. Canopy Volume Estimation Using Target Values Converted from Canopy Fullness Levels

**Figure 9:**
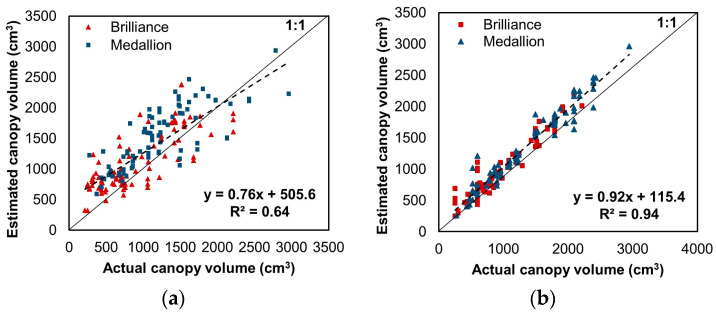
Comparison of canopy volumes estimated from the developed model and the actual canopy volumes measured using EPS balls, (**a**) when 100% of canopy volume converted from canopy fullness level was used as target value and (**b**) when a 50/50 mix of converted canopy volume and canopy volume measured using EPS balls was used as target value. The color indices denote the values for each strawberry variety. The dashed lines show the regression lines.

Figure 9a shows the outcome of the CNN model trained with canopy volume converted from the canopy fullness level compared to the actual canopy volume measured using EPS balls. A linear relationship was observed, with an R^2^ and RMSE value of 0.64 and 421.2 cm^3^, respectively. Despite the decreased estimation performance, the model showed improved results compared to the linear regression model using the paraboloid. The effectiveness of fine-tuning the developed model using canopy fullness level was assessed with the model trained with a 50/50 mix of the measured canopy volumes using EPS balls and canopy volume converted from the canopy fullness level. In this case, the model achieved enhanced estimation results, as illustrated in Figure 9b, with an R^2^ of 0.94 and RMSE of 141.3 cm^3^. Although the result recorded a slight increase in RMSE, the performance was insignificantly degraded compared to the model trained through canopy volume measured using EPS balls only.

### 3.3. Distributions of Estimated Canopy Volume Using CNN

**Figure 10:**
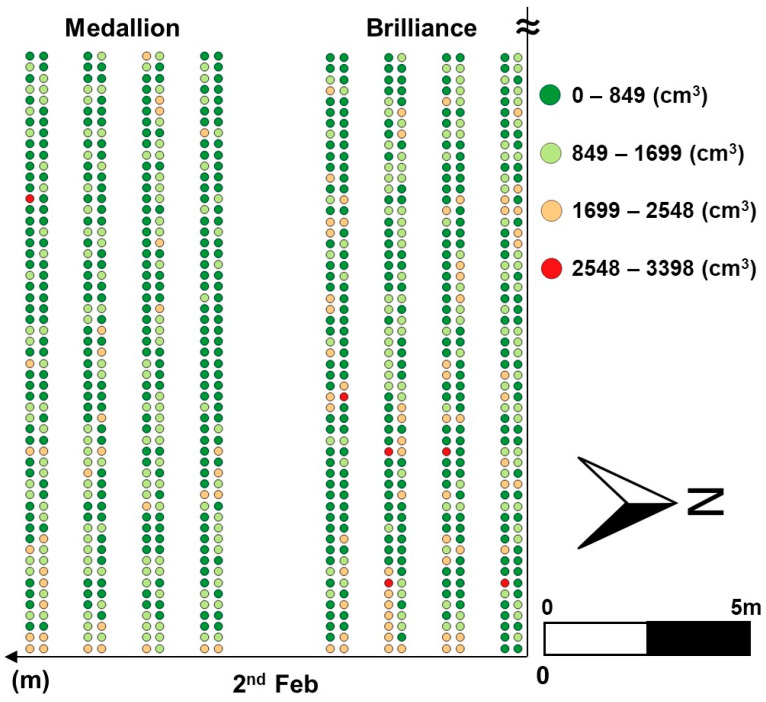
A part of the canopy volume distribution map in the entire field.

**Figure 11:**
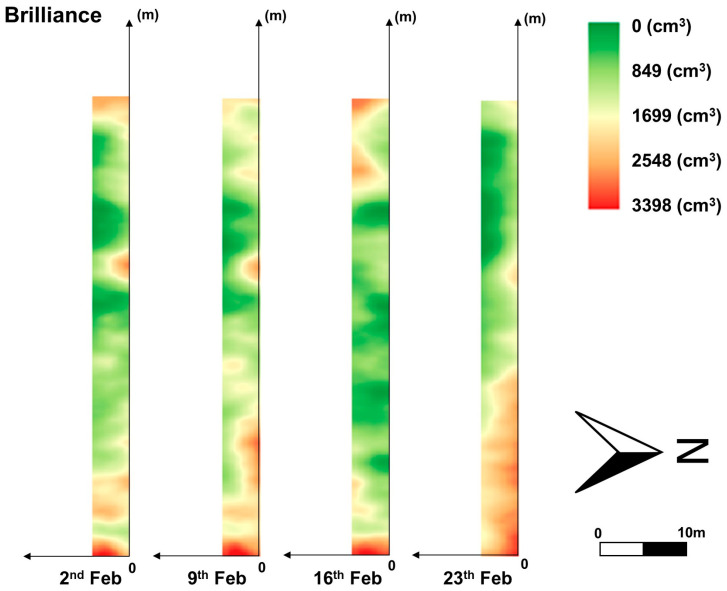
Canopy volume distribution of the Brilliance variety from 2 to 23 February 2024.

**Figure 12:**
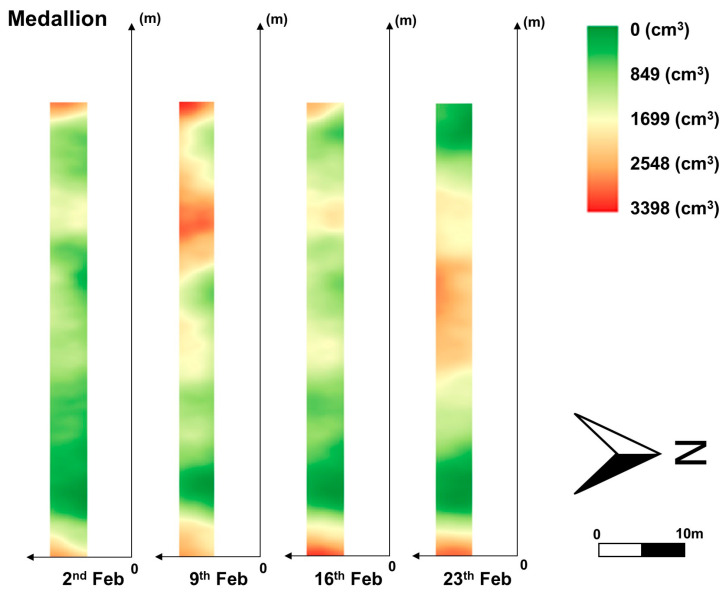
Canopy volume distribution of the Medallion variety from 2 to 23 February 2024.

A continuous combination of images from the UAV was achieved despite image distortions caused by left and right biases during manual drone flight over some sections of the field. The row images were divided and processed again into the YOLOv8n model to extract individual plants. Finally, canopy volume for these cropped plants was estimated using the CNN model, and the results were interpolated using regular kriging to generate approximate canopy volume distributions, as presented in Figure 10, Figure 11 and Figure 12. Figure 10 portrays a part of the canopy volume distribution on 2 February. Contour maps were also added in Figure 11 and Figure 12 to easily identify spatial differences in canopy volume, which highlighted the actual canopy volume sizes. These results indicate the potential use of the developed models for growth monitoring and canopy volume estimation across the entire strawberry field.

**Figure 13:**
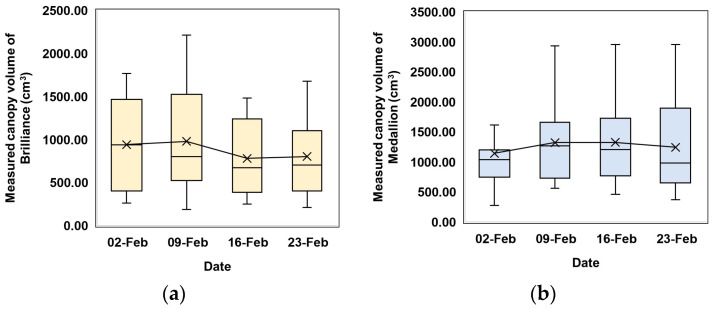
Box plots of weekly canopy volumes of the sampled plants measured using the EPS balls: (**a**) Brilliance variety; (**b**) Medallion variety for 40 sampled plants.

The canopy volume of the Brilliance variety in the east of the field increased over time. However, the canopy volume of the Medallion variety in the east of the field remained at a similar level over the same period. This result indicates that the canopy volume distributions of the two cultivars are different and should be managed individually. The canopy volume in both varieties increased by the second week. The Brilliance variety showed a noticeable decline in canopy volume on 16 February, while the Medallion variety recorded a slight reduction on the same day. By 23 February, both varieties exhibited fluctuating growth trends. Canopy volumes increased until the second week and then decreased, as illustrated in Figure 11 and Figure 12. These changes in canopy volume observed in the distribution maps correspond to those measured using EPS balls for 40 sampled plants, as shown in Figure 13. These similarities suggest that the distribution maps based on the models developed in this study can effectively provide an overview of canopy volumes across the entire strawberry field.

## 4. Discussion

Numerous studies have employed simple geometry for easy canopy volume estimations [10,13,14,15,16]. Notably, the CNN model developed in this present study significantly improved estimation performance compared to linear regression using the paraboloid. Moreover, the proposed model applied simple image acquisition devices and affordable computational processes compared to past methods that relied on complex computational geometry and voxelization [5,10,22,24,25,28,29]. The CNN-based model also exhibited high accuracy and fast inference speeds, which can be operated using embedded boards, as described in earlier research [34].

CNNs use small filters to extract local features from images, enabling the model to focus on essential regions. CNNs are effective at identifying crucial details by learning from basic features, such as edges, or more complex features, for example, shapes. These capabilities allow CNNs to accurately estimate canopy volume from the morphological features of crops, even when processing complex and nonlinear relationships [61].

With appropriate target values, CNNs can potentially estimate the internal space of a strawberry canopy based on RGB image features. Previously, Zheng et al. used a CNN model to estimate canopy volume from RGB, NIR, DSM, and mask images from UAV images. Despite a high R^2^ value of 0.927, the model was trained using target values obtained from grid cell-based calculations via Spatial Analyst in ArcMap 10.7 software (ESRI, Redlands, CA, USA), which may introduce inaccurate volume estimation. In addition, canopy volume distribution was not constructed for the entire field [7,42]. Reducing the number of sensors is also essential to enhance the model’s applicability to agricultural tasks.

Canopy volume estimation performance can be improved by measuring the target canopy volumes accurately. Volume measurement can be obtained by calculating the difference between the volume of the space containing the object and the volume of the space without the object. In one study, Andújar et al. used a water displacement method to measure fruit volumes [62], similar to the approach used in this study. The generation of 3D models of cauliflower fruits was achieved using a depth camera for volume estimation, which recorded an R^2^ and RMSE of 0.87 and 19.57 cm^3^, respectively. Unlike strawberry plants, cauliflower fruits do not require internal structure consideration, so the error can be minimal. Meanwhile, constructing their 3D model requires a 360° sensor rotation and complex processing. In contrast, the model developed in this study achieved high accuracy and low RMSE for canopy volume estimation using only top-view RGB images despite accounting for the internal space of the strawberry canopy.

Image augmentation could further enhance the CNN model accuracy using multiple images per plant extracted from video snapshots during training. The application of the YOLOv8n object detection model to adjust for image size due to manual UAV flight altitude variations also reduced errors associated with canopy volume estimation. Although image registration algorithms can minimize image scale variations due to altitude changes during manual UAV flight [44,45,46], they require massive computational resources and can lead to misaligned composite images in complex vegetation scenes without GNSS information. Conversely, object detection-based image synthesis simplified the image-combining process and prevented aligning failures.

The estimation errors increased as the strawberry plant grew in size, leading to a higher overall RMSE in later growth stages. These errors may be due to the sole reliance on RGB images during estimation without any vertical orientation information of the plants [34], as well as a relatively small number of large-canopy plant samples. However, a management plan can be established by focusing on plants that exceed a certain canopy size threshold, where the accuracy of canopy volume estimation above the threshold may be less critical.

Furthermore, using canopy volume converted from canopy fullness level as a target value for the CNN model proved effective for canopy volume estimation, particularly when combined with canopy volume measured using EPS balls. The 50/50 mixture enhanced performance by enabling the CNN to learn both general patterns and detailed information. While converted canopy volume data can lead to some information loss, continuous data can mitigate the loss and reduce performance degradation. Therefore, canopy fullness data may be used to significantly shorten the time needed to acquire canopy volume estimation and improve model fine-tuning under varying environments in the future.

Deep learning and UAV technologies have been effective in precision agriculture, particularly for monitoring and managing individual plants [33,39,40,41,42,63]. Combining both in this study, the developed CNN model showed its ability to assess UAV images of the entire strawberry field with object detection for canopy volume distribution. The results may facilitate enhancing the allocation of labor and resource management, including during harvesting [64,65,66]. UAVs equipped with RGB cameras and without GNSS offer an economical and easy-to-operate approach for virtual growth monitoring, providing a valuable alternative solution for estimating field images and crop growth.

Although improving the accuracy of object detection and minimizing detection failures remain critical challenges that warrant further assessment, the detection metrics for both strawberries and missing plants were high in this study, consistent with previous research on strawberries [67,68] and the use of YOLOv8n [50]. For labels other than strawberries, no plants were present in all instances. Consequently, any detection, regardless of label, could be used to construct a virtual growth map.

## 5. Conclusions

This study successfully developed and evaluated a CNN model to estimate canopy volumes of field-grown strawberries in Florida using UAV-acquired RGB images. Object detection-based image processing methods were introduced to correct for changes in manual UAV flight altitudes and generate row-wide images without relying on GNSS information. Canopy measurement data were obtained using EPS balls and an acrylic case to determine the internal structure of the plants. Furthermore, training with canopy volume data converted from canopy fullness was effective, confirming the potential to fine-tune with canopy fullness levels. Since canopy volumes may be related to crop yield, harvest timing, and various management practices, such as irrigation, disease, and pest control, the developed method offers farmers a practical field monitoring approach and aids in efficient labor and resource allocation without wasting essential time and vital resources. Finally, the proposed model may contribute to improving strawberry productivity, with potential application extended to other various low-height crops. This method can also be useful in monitoring the health and growth of forestry and horticultural pants, and for wildfire risk management. It is noteworthy that future studies should focus on developing models based on the results from this study and canopy volume to predict actual strawberry yield. In addition, this estimation model will be installed in an embedded system for real-time growth predictions to improve the convenience of image-based growth measurements.

## Figures and Tables

**Table 1:** Detailed specifications of the acrylic cylindrical case, EPS balls, and the resulting volume measurements using this method.

Specifications	Values
Inner diameter of the cylindrical case	19.4 cm
Height of the cylindrical case	10.1 cm
Volume of the cylindrical case	2985.5 cm^2^
Diameter of an EPS ball	2 cm
Volume of an EPS ball	4.19 cm^3^
Total number of EPS balls filling the case	240 pieces
Total volume of 240 EPS balls	1005.6 cm^3^
Space not filled by EPS balls (offset)	1979.9 cm^3^
Offset per ball	8.25 cm^3^

**Table 2:** Object detection performance of the trained YOLOv8n model.

Object	mAP50	Precision (%)	Recall (%)	F1-Score	Average Frames per Second
All	0.78	89.7	88.7	0.89	18.6
Strawberry	0.98	94.4	97.9	0.96	-
Dead plant	0.74	62.2	74.2	0.68	-
Hole (missing plant)	0.90	86.9	96.3	0.91	-
Weed	0.63	47.6	50.0	0.49	-

## Data Availability

The datasets presented in this article are not readily available because the data are part of an ongoing study.
